# Biliary Tract Carcinogenesis Model Based on Bile Metaproteomics

**DOI:** 10.3389/fonc.2020.01032

**Published:** 2020-07-24

**Authors:** Ariel A. Arteta, Miryan Sánchez-Jiménez, Diego F. Dávila, Oscar G. Palacios, Nora Cardona-Castro

**Affiliations:** ^1^School of Graduate Studies, CES University, Medellín, Colombia; ^2^Basic Science Research Group, School of Medicine, CES University, Medellín, Colombia; ^3^Associated Professor Department of Pathology, University of Antioquia, Medellín, Colombia; ^4^Colombian Institute of Tropical Medicine (ICMT), Sabaneta, Colombia; ^5^Department of Hepatobiliary and Pancreatic Surgery, CES Clinic, Medellín, Colombia

**Keywords:** pancreatic cancer, metaproteomic, proteomic, bile, zeatin, surfactin, IL-8, carcinogenesis model

## Abstract

**Purpose:** To analyze human and bacteria proteomic profiles in bile, exposed to a tumor vs. non-tumor microenvironment, in order to identify differences between these conditions, which may contribute to a better understanding of pancreatic carcinogenesis.

**Patients and Methods:** Using liquid chromatography and mass spectrometry, human and bacterial proteomic profiles of a total of 20 bile samples (7 from gallstone (GS) patients, and 13 from pancreatic head ductal adenocarcinoma (PDAC) patients) that were collected during surgery and taken directly from the gallbladder, were compared. g:Profiler and KEGG (Kyoto Encyclopedia of Genes and Genomes) Mapper Reconstruct Pathway were used as the main comparative platform focusing on over-represented biological pathways among human proteins and interaction pathways among bacterial proteins.

**Results:** Three bacterial infection pathways were over-represented in the human PDAC group of proteins. IL-8 is the only human protein that coincides in the three pathways and this protein is only present in the PDAC group. Quantitative and qualitative differences in bacterial proteins suggest a dysbiotic microenvironment in the PDAC group, supported by significant participation of antibiotic biosynthesis enzymes. Prokaryotes interaction signaling pathways highlight the presence of zeatin in the GS group and surfactin in the PDAC group, the former in the metabolism of terpenoids and polyketides, and the latter in both metabolisms of terpenoids, polyketides and quorum sensing. Based on our findings, we propose a bacterial-induced carcinogenesis model for the biliary tract.

**Conclusion:** To the best of our knowledge this is the first study with the aim of comparing human and bacterial bile proteins in a tumor vs. non-tumor microenvironment. We proposed a new carcinogenesis model for the biliary tract based on bile metaproteomic findings. Our results suggest that bacteria may be key players in biliary tract carcinogenesis, in a long-lasting dysbiotic and epithelially harmful microenvironment, in which specific bacterial species' biofilm formation is of utmost importance. Our finding should be further explored in future using *in vitro* and *in vivo* investigations.

## Introduction

Pancreatic ductal adenocarcinoma (PDAC) is a malignant and highly lethal neoplasm of unknown etiology and is usually diagnosed at advanced stages (1). The currently available surgical interventions and chemotherapeutic regimes are unable to provide the desired impact on disease outcomes, and there is a clear, dismal prognosis, as 70–80% of patients will succumb to this disease during the first 2 years post-diagnosis (2). PDAC is the fourth leading cause of cancer-related deaths worldwide (3) and is expected to become the second leading cause of cancer-related deaths by 2025, due both to the improved outcome of other malignancies, and on the stagnation in outcome improvement for PDAC over the past 30 years (4–6). Modifiable and non-modifiable risk factors for PDAC have an unconvincing molecular association with the disease. Modifiable factors seem to distribute haphazardly around the world, and the classic ones, such as tobacco, diabetes, gallstones (GS) and alcohol intake, are absent in a significant proportion of patients (7, 8). The development of interventions that successfully reduce the incidence of this lethal malignancy and improve its outcome is limited by the scarce knowledge of the molecular factors that may play a role in the complex process of PDAC carcinogenesis (9). Hence, any effort to better understand PDAC carcinogenesis, or to unravel novel therapies, may be the starting point in driving future clinical interventions.

Bacteria have been associated with benign and malignant disease, and bacterial carcinogenesis is a process still being characterized in detail. The knowledge from such study may be the starting point to drive clinical interventions focused on cancer prevention. The carcinogenesis associated with viruses is based on the integration of the viral genome into the host DNA (i.e., Human Papilloma Virus, Epstein-Barr) and has been extensively studied and characterized (10). Conversely, bacterial carcinogenesis is a phenomenon thought to be the result of epithelial cells' chronic exposure to a pro-inflammatory milieu exacerbated by bacteria (11, 12). However, this pro-inflammatory, physiopathological mechanism cannot explain convincingly by itself the development of carcinomas in the gastrointestinal and biliary tract, as inflammatory phenomena regularly occur throughout the human lifespan, and just a few human beings develop malignant neoplasms.

The biliary tract including intra-pancreatic bile ducts, is a semi-closed duct system possessing its own microbiota (13–15), lined by cholangiocytes, and in constant contact with bile. Cholangiocytes or cholangiocyte like cells are the proposed cell of origin for a range of biliary tract carcinomas, also named cholangiocarcinoma, in gallbladder, and intra or extrahepatic bile ducts (16). PDAC derives from ductal cholangiocytes or transdifferentiated acinar-to-ductal cholangiocytes (17, 18) covering intra-pancreatic bile ducts (ductal carcinoma), so from the histopathological point of view PDAC and biliary tract carcinomas are not very different (19). In the case of PDAC, local microbiota may have effects on oncogenesis (20) and long term survival (21), but most of the surveys associating PDAC and bacteria demonstrate spurious associations due to inconsistent isolation of specific bacterial species and the lack of a molecular basis for bacteria-induced carcinogenesis (22, 23). Being part of the biliary tract microenvironment, bacteria must contribute to bile protein pool composition in a similar way to cholangiocytes. As cholangiocytes and bacteria are in permanent contact with bile, we hypothesize that bile-associated protein changes could reflect bile duct system alterations in the microenvironment sufficient to transform benign epithelial cells into a malignant phenotype.

Bile is stored and concentrated in the gallbladder, which is a clean reservoir where this biological fluid can be extracted for protein analysis (24). In research, bile samples are typically taken from the distal portion of the biliary tract during endoscopic interventions, such as endoscopic retrograde cholangiopancreatography (ERCP) (25). However, the inflammatory process associated with biliary obstruction in most PDAC patients may alter bile protein composition in the distal portion of the biliary tract and limit the finding of meaningful biological information. The lack of meaningful biological findings hinders the development of a specific model of carcinogenesis for the biliary tract that takes into account its unique physiological conditions, and the interplay of human and bacterial proteins.

We analyzed samples of human bile taken directly from the gallbladder, and not by ERCP, exposed to a pancreatic tumor vs. non-tumor microenvironment. The aim of the study, once samples were analyzed by mass spectrometry, was to find meaningful biological information through pathway inference analysis of the identified human and bacterial proteins. Biological pathway analysis was initially performed using the g:Profiler platform to compare and generate a complete panorama of the gene-protein sets being analyzed, including over-represented KEGG biological pathways (26). Then, we directly analyzed each protein set using the KEGG Mapper Reconstruct Pathway module (27), focusing on over-represented pathways in g:Profiler for human proteins, and prokaryote interaction pathways for bacterial proteins. KEGG has become a world reference database for assisting biological interpretations of molecular data sets. Currently, biological pathway analyses are one of the most reliable strategies for mechanistic insights into omics data, since the kind of evidence that supports the statistical modeling is always experimental and manually curated (28). Thus, in this study, using a paradigm shifting metaproteomic approach, we aimed to unravel novel and meaningful biological information that may contribute to a better understanding of PDAC bacteria-induced carcinogenesis, proposing a new carcinogenesis model for the biliary tract.

## Materials and Methods

### Ethics and Sample Acquisition

The Institutional Human Ethics Committee at CES University and Clinic approved this study, and patients must give informed consent. Samples were de-identified before performing proteomic analysis. A surgical pathologist collected a total of 20 gallbladder bile samples; seven from patients with gallstones (GS), and 13 from patients with PDAC arising from the head of the pancreas. All patients were Colombians, and residents of Medellín (Colombia). For GS patients, bile was obtained in the operating room immediately after laparoscopic extraction of the gallbladder, puncturing the gallbladder fundus with a syringe, and aspirating at least 5 mL of bile. For PDAC patients, bile was similarly collected, by aspirating bile with a syringe from the gallbladder pancreatoduodenectomy specimens were sent to the pathology lab for a cryosection margin report. Immediately after collection, bile samples were transported on ice, aliquoted, and stored at −80°C until further analysis. Patients with a clinical history of previous malignant neoplasms, chemotherapy, prior biliary tract surgery or biliary stent placement, HIV, pregnancy, chronic pancreatitis, choledocholithiasis, cystic fibrosis, hepatolithiasis, primary biliary cholangitis, liver cirrhosis, primary sclerosing cholangitis, or acute cholecystitis were excluded from this study.

### Protein Extraction

Bile samples were thawed at room temperature and processed as previously described with slight modifications (29). Briefly, 1 mL of bile was centrifuged for 10 min at 4°C and 3,000 rpm, and 1 mL of TRI reagent and 1 mL of chloroform were added. The mix was incubated for 5 min at room temperature (20–25°C) and centrifuged for 15 min at 4°C and 12.000 xg to separate proteins. Avoiding the central lipid layer, remaining tube contents (supernatant + pellet) were transferred to a new tube. Then, 1,200 μL of acetone was added, mixed, incubated for 4 h, and centrifuged for 15 min at 4°C at 12,000 xg. Acetone was discarded, and the tubes were dried at room temperature, after which 200 μL of reconstituting buffer was added to the pellet, and the solution dried and lyophilized.

### Proteomic Analyses

Proteomic analysis was performed by Creative Proteomics (Ramsey Road, Shirley, NY 11967, USA), briefly, the techniques used are described as follows:

#### Sample Preparation for Proteomic Analysis

Total proteins were precipitated from the protein solution using methanol and chloroform. Approximately 10 μg of total protein was dissolved in 6 M urea aqueous solution and was denatured with 10 mM DL-dithiothreitol, incubated at 56°C for 1 h, followed by alkylation with 50 mM iodoacetamide, and incubated for 60 min at room temperature, protected from light. Next, 500 mM ammonium bicarbonate (ABC) was added to the solution to make a final concentration of 50 mM ABC with a pH of 7.8. Promega Trypsin was added to the protein solution for digestion at 37°C for 15 h. The generated peptides were further purified with the C18 SPE column (Thermo Scientific) to remove salt. Samples were dried in a vacufuge and stored at −20°C until use.

#### Nano Liquid Chromatography

An Easy-nLC1000 (ThermoFisher Scientific, USA) coupled to a 100 μm × 10 cm in-house made column packed with a reversed-phase ReproSil-Pur C18-AQ resin (3 μm, 120 Å, Dr. Maisch GmbH, Germany) was used. A sample volume of 5 μL was loaded, with a total flow rate of 600 nL/min, and a mobile phase of A: 0.1% formic acid in water; and B: 0.1% formic acid in acetonitrile. The analytical separation was run using a gradient: from 6 to 9% B for 15 min, from 9 to 14% B for 20 min, from 14 to 30% B for 60 min, from 30 to 40% B for 15 min and from 40 to 95% B for 3 min, eluting with 95% B for 7 min.

#### Mass Spectrometry and Data Analysis

An Orbitrap Q Exactive™ mass spectrometer (Thermo Fisher Scientific, USA) set on a spray voltage of 2.2 kV and a capillary temperature of 270°C was used. Mass spectrometry resolution was set to 70,000 at 400 m/z and precursor m/z range: between 300.0 and 1800.0. The production scan range starts from m/z 100, activated by collision-induced dissociation (CID), and an isolation width of 3.00. The raw files were analyzed and searched against the human protein database from Uniprot using Maxquant (1.5.6.5). The parameters were set as follows: the protein modifications were carbamidomethylation (C) (fixed), oxidation (M) (variable); the enzyme specificity was set to trypsin; the maximum missed cleavages was set to 2; the precursor ion mass tolerance was set to 10 ppm, and MS/MS tolerance was 0.6 Da.

### Human and Bacteria Peptide-Protein List Selection for Analysis

Peptide-protein analysis was performed at ICMT-CES University. Contaminants, albumin, hemoglobin related peptides, and peptides with zero intensity were eliminated from the full human and bacteria list of peptides-proteins. The identifiers of protein were standardized, missing gene names were manually completed, and protein taxonomy was verified.

Then, the full list of shared proteins was adapted to meet the requirements of the Prostar platform online version 1.18.1 (30), seeking for differentially abundant human and bacterial proteins among groups (GS vs. PDAC). The intensity values were normalized with the mean centering method without including variance reduction. Partially observed values were imputed using the SLSA (Structured Least Squares Adaptive) method. The hypothesis test was performed using the Student's *t*-test, considering a logarithmic change of 2.5 and adjusting the false discovery rate to 0.42% (*p*-value = 0.00316). The biological validity of imputing non-existent values for non-observed proteins, in order to compare the exclusive groups of proteins, was explored. However we chose to perform the analysis based only on observed values in the two groups, GS and PDAC (shared proteins).

**Figure 1:**
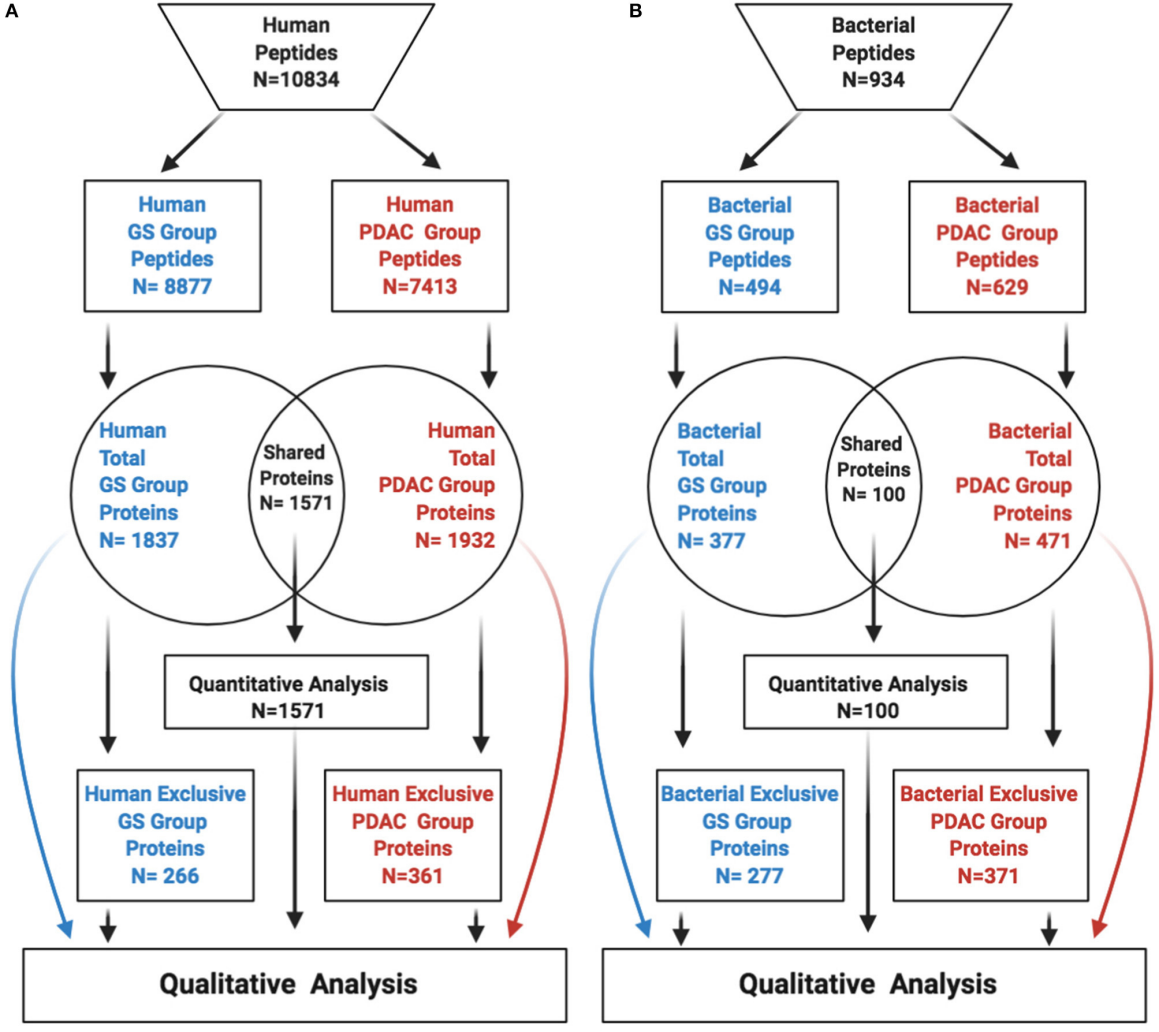
Identified peptides-proteins per group and origin. The figure depicts peptides-proteins identified from human **(A)**, and bacteria **(B)**. GS, gallstones; PDAC, pancreatic ductal adenocarcinoma.

For further qualitative analysis, all the human protein lists of the total, exclusive and differentially abundant proteins from GS and PDAC patients (Figure 1) were included in the retrieve ID/mapping module of the Universal Protein consortium resource (Uniprot http://www.uniprot.org/, UniProt release 2019_10). Then, in order to provide mechanistic insights into the biologically integrated function, Uniprot-standardized human protein lists of entries for each group were analyzed in the g:Profiler web page (https://biit.cs.ut.ee/gprofiler/gost) (26). g:Profiler allows a multi-query approach, which performs an over-representative functional analysis of multiple protein-gene lists, comparing proteins among groups. Default options were maintained in g:Profiler, adding no electronic gene ontology annotations, and Bonferroni correction for multiple test adjustments. Significant, adjusted, over-represented pathways (*p*-values < 0.01), were used for further analysis in the KEGG Mapper Reconstruct Pathway. KEGG identifiers were obtained from the Uniprot FASTA file of the total, exclusive, and differentially abundant protein list, through BlastKoala (KEGG Orthology and Links Annotation version 2.2 https://www.kegg.jp/blastkoala/) (31). KEGG Mapper Reconstruct Pathway allows visualization and comparison of proteins in signaling pathways to identify qualitative and quantitative differences without coupled statistical analysis. (https://www.genome.jp/kegg/tool/map_pathway.html) (27). On the other hand, useful drugs were explored using the functional database DrugBank through WebGestalt (WEB-based GEne SeT AnaLysis Toolkit updated on 01/14/2019 http://www.webgestalt.org/) (32), by performing an over-representation analysis (ORA), using the database Drugbank and setting the false discovery rate at <0.01 (32) with Bonferroni correction for multiple test adjustments.

**Figure 2:**
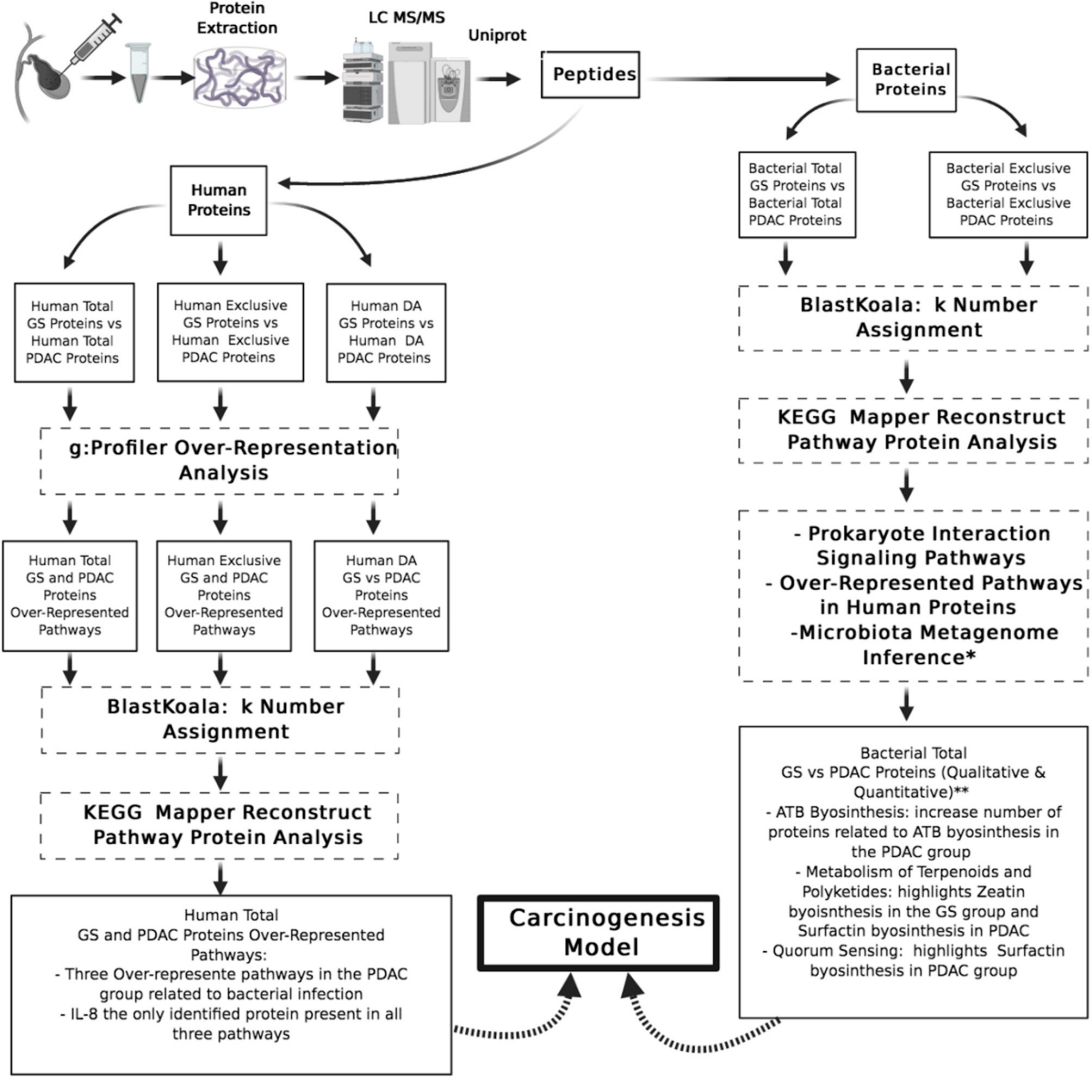
Pipeline of proteins data sets analysis and summary or relevant findings. GS, gallstones; PDAC, pancreatic ductal adenocarcinoma; DA, differentially abundant; KEGG, Kyoto encyclopedia of genes and genomes; IL-8, interleukin 8; ATB, antibiotics. *Small sample microbiota metagenome inference analysis (unpublished results). **Not statistical analysis associated.

The biological context was analyzed as a whole for the total protein groups by correlating findings with the specific proteins identified for each condition. Many biological pathways were enriched over the Bonferroni p-adjusted value threshold in the total protein groups, but just three of them were related to bacterial infection (Figure 2).

For bacterial proteins, the same protocol for contaminant elimination, quality control, and differential abundance analysis was performed as for human proteins. We did not use g:Profiler for bacterial protein analysis because this platform is not conceived for multi-species analysis. The bacterial protein lists of total and exclusive proteins from GS and PDAC patients were also included in the retrieve ID/mapping module of the Universal Protein consortium resource. KEGG identifiers were obtained using BlastKoala from the Uniprot FASTA files, and we focused our attention on prokaryote interaction signaling pathways during the analysis in KEGG Mapper Reconstruct Pathway (33).

## Results

A total of 20 bile samples extracted from gallbladders were analyzed, seven of which were taken from patients with GS (mean age of 48 years) (Table 1), and 13 from patients with PDAC (mean age of 56 years). All the patients were residents in Medellín and none of the patients presented clinical or histopathological signs of acute inflammation.

**Table 1:** Clinical and demographic characteristics of patients.

**Diagnosis**	**Age**	**Sex**	**Gallstones**	**Pathologic Staging**	**Perineural Invasion**
GS	66	M	(+)	NA	NA
GS	52	F	(+)	NA	NA
GS	32	F	(+)	NA	NA
GS	42	M	(+)	NA	NA
GS	55	F	(+)	NA	NA
GS	48	M	(+)	NA	NA
GS	46	F	(+)	NA	NA
PDAC	52	F	(+)	pT2N0	(+)
PDAC	58	M	(+)	pT3N1	(+)
PDAC	66	M	(-)	pT2N1	(+)
PDAC	46	M	(-)	pT3N1	(+)
PDAC	68	M	(-)	pT3N1	(+)
PDAC	60	M	(-)	pT3N1	(+)
PDAC	35	M	(-)	pT2N1	(+)
PDAC	60	F	(+)	pT2N0	(+)
PDAC	56	F	(-)	pT3N0	(+)
PDAC	58	M	(-)	pT3N1	(+)
PDAC	61	F	(+)	pT2N1	(+)
PDAC	62	F	(-)	pT3N0	(+)
PDAC	55	M	(-)	pT3N1	(+)

*GS, gallstones; PDAC, pancreatic ductal adenocarcinoma; M, male; F, female*.

After excluding peptides that were unassociated with any known proteins, a total of 10,834 human peptides were identified with a mean of 542 peptides per sample, 8,877 peptides in the GS group and 7,413 in the PDAC group. Peptides were associated with a total of 2,198 human proteins, 1,837 proteins in the GS group, and 1,932 proteins in the PDAC group. Upon comparison, a total of 1,571 proteins were common to both groups, while 266 proteins were exclusively found in the GS group, and 361 proteins in the PDAC group (Figure 1A). For bacteria, we identified a total of 934 peptides with a mean of 46 peptides per sample, 494 in the GS group and 629 in the PDAC group. Those peptides were associated with a total of 748 bacterial proteins, 377 proteins in the GS group and 471 in the PDAC group. We found 100 proteins shared among the two groups, with 277 exclusive proteins remaining in the GS group and 371 in the PDAC group (Figure 1B). Quantitative differential abundance analysis using Prostar revealed among the shared proteins within the human and bacteria groups, 123 differentially abundant human proteins, 81 in the GS group and 42 in the PDAC group, and no differentially abundant bacterial proteins.

### Human Protein Over-Representation Analysis

The g:Profiler platform was used for the over-representation analysis in KEGG signaling pathways. Analyzing the total list of proteins, the platform identified from the 1,837 proteins in the GS group 1,832 (99.7%) and from 1,932 in the PDAC group 1,929 (99.8%). Regarding exclusive and differentially abundant proteins, the platform identified 100% of proteins in the GS and PDAC groups. In the total protein lists, we found five over-represented pathways in the GS group and seven in the PDAC group (Table 2), and in the exclusive protein lists, we identified one over-represented pathway in each group: phagosome in the GS group and metabolic pathways in the PDAC group. The analysis of the differentially abundant list of proteins revealed just one over-represented pathway in the GS group: vasopressin-regulated water reabsorption.

**Table 2:** g:Profiler over-represented signaling pathway in human proteins.

**Total Proteins GS** **=** **1837 PDAC** **=** **1932**			
**Term**	**ID**	***p*****-Adjusted value GS**	***p*****-Adjusted value PDAC**
Pertussis	KEGG:05133	0.23886	0.00061
Proximal tubule bicarbonate reclamation	KEGG:04964	0.00094	0.06026
Cholesterol metabolism	KEGG:04979	0.03619	0.00105
Drug metabolism—other enzymes	KEGG:00983	0.01750	0.00111
Amino sugar and nucleotide sugar metabolism	KEGG:00520	0.00116	0.03220
Sulfur metabolism	KEGG:00920	0.00196	0.04153
Glyoxylate and dicarboxylate metabolism	KEGG:00630	0.03702	0.00203
Legionellosis	KEGG:05134	0.01740	0.00231
Adherens junction	KEGG:04520	0.00280	0.05119
Arginine and proline metabolism	KEGG:00330	0.02670	0.00305
Shigellosis	KEGG:05131	0.01019	0.00575
**Exclusive Proteins GS** **=** **266 PDAC** **=** **361**			
Phagosome	KEGG:04145	0.00011	0.04876
Metabolic pathways	KEGG:01100	1	0.00142
**Differentially Abundant Proteins GS** **=** **81 PDAC** **=** **42**			
Vasopressin-regulated water reabsorption	KEGG:04962	0.00066	1

*KEGG, Kyoto Encyclopedia of Genes and Genomes; GS, gallstones; PDAC, pancreatic ductal adenocarcinoma*.

The over-represented pathways were analyzed in KEGG Mapper Reconstruct Pathway focusing our attention in the three g:Profiler over-represented pathways in the PDAC total protein group related to the bacterial infections Shigellosis, Pertussis, and Legionellosis. Analyzing the list of proteins in these three pathways (Table 3), it is notable that IL-8 (interleukin 8) is the only protein coinciding in the three pathways and present only in the PDAC group. This difference is more remarkable when evaluating the pathways of cytokine-cytokine receptor interaction and cytokines and growth factors, the latter in BRITE (Functional hierarchies of biological entities) tables, finding association in the presence of IL-8 with interleukin 11 (IL-11), CCL15 (Chemokine (C-C motif) ligand 15), CSF1 (Macrophage colony-stimulating factor) and CXCL7 (Chemokine (C-X-C motif) ligand 7) in the PDAC group (Table 4). Considering as an interaction point among prokaryotes and eukaryotes, Toll-like and NOD-like receptor pathways, we analyzed those signaling pathways and IL-8 was also present, and only in the PDAC group. In other KEGG signaling pathways with relevance to carcinogenesis processes such as DNA repair, xenobiotic metabolism, and pathways in cancer and pancreatic cancer, we didn't find differences. In the signaling pathways over-represented in exclusive and differentially abundant proteins, we could not find a clear biological meaning. In the WebGestal platform analysis, there were no relevant results for useful, therapeutic drugs using the Drugbank database.

**Table 3:** Human total proteins per group in KEGG of bacterial infection over-represented signaling pathways.

**Shigellosis** ***N*** **=** **20**			**Pertussis** ***N*** **=** **22**			**Legionellosis** ***N*** **=** **21**		
**Term**	**Condition**	**Protein**	**Term**	**Condition**	**Protein**	**Term**	**Condition**	**Protein**
K04371	Gallstones:	MK01_HUMAN	K01330	Gallstones:	C1R_HUMAN	K01370	Gallstones:	
	Carcinoma:	MK03_HUMAN		Carcinoma:	C1R_HUMAN		Carcinoma:	CASP1_HUMAN
K04392	Gallstones:	RAC1_HUMAN	K01331	Gallstones:	C1S_HUMAN	K03231	Gallstones:	EF1A2_HUMAN
	Carcinoma:	RAC1_HUMAN		Carcinoma:	C1S_HUMAN		Carcinoma:	EF1A2_HUMAN
K04393	Gallstones:	CDC42_HUMAN	K01332	Gallstones:	CO2_HUMAN	K03233	Gallstones:	EF1G_HUMAN
	Carcinoma:	CDC42_HUMAN		Carcinoma:	CO2_HUMAN		Carcinoma:	EF1G_HUMAN
K04438	Gallstones:	CRK_HUMAN	K01370	Gallstones:		K03283	Gallstones:	HS71B_HUMAN
	Carcinoma:	CRK_HUMAN		Carcinoma:	CASP1_HUMAN		Carcinoma:	HS71B_HUMAN
K04514	Gallstones:		K02183	Gallstones:	CALM3_HUMAN	K03990	Gallstones:	CO3_HUMAN
	Carcinoma:	ROCK1_HUMAN		Carcinoma:	CALM3_HUMAN		Carcinoma:	CO3_HUMAN
K05692	Gallstones:	ACTB_HUMAN	K03986	Gallstones:	C1QA_HUMAN	K04077	Gallstones:	CH60_HUMAN
	Carcinoma:	ACTB_HUMAN		Carcinoma:	C1QA_HUMAN		Carcinoma:	CH60_HUMAN
K05700	Gallstones:	VINC_HUMAN	K03987	Gallstones:	C1QB_HUMAN	K04391	Gallstones:	CD14_HUMAN
	Carcinoma:	VINC_HUMAN		Carcinoma:	C1QB_HUMAN		Carcinoma:	CD14_HUMAN
K05748	Gallstones:	WASF2_HUMAN	K03988	Gallstones:	C1QC_HUMAN	K05482	Gallstones:	IL18_HUMAN
	Carcinoma:			Carcinoma:	C1QC_HUMAN		Carcinoma:	IL18_HUMAN
K05754	Gallstones:	ARPC5_HUMAN	K03989	Gallstones:	CO4A_HUMAN	K06461	Gallstones:	ITAM_HUMAN
	Carcinoma:	ARPC5_HUMAN		Carcinoma:	CO4A_HUMAN		Carcinoma:	ITAM_HUMAN
K05755	Gallstones:	ARPC4_HUMAN	K03990	Gallstones:	CO3_HUMAN	K06464	Gallstones:	
	Carcinoma:	ARPC4_HUMAN		Carcinoma:	CO3_HUMAN		Carcinoma:	ITB2_HUMAN
K05756	Gallstones:	ARPC3_HUMAN	K03994	Gallstones:	CO5_HUMAN	K07874	Gallstones:	RAB1A_HUMAN
	Carcinoma:	ARPC3_HUMAN		Carcinoma:	CO5_HUMAN		Carcinoma:	RAB1A_HUMAN
K05757	Gallstones:	ARC1B_HUMAN	K04001	Gallstones:	IC1_HUMAN	K07875	Gallstones:	RAB1B_HUMAN
	Carcinoma:	ARC1B_HUMAN		Carcinoma:	IC1_HUMAN		Carcinoma:	RAB1B_HUMAN
K05758	Gallstones:	ARPC2_HUMAN	K04002	Gallstones:	C4BPA_HUMAN	K07937	Gallstones:	ARF1_HUMAN
	Carcinoma:	ARPC2_HUMAN		Carcinoma:	C4BPA_HUMAN		Carcinoma:	ARF1_HUMAN
K05759	Gallstones:	PROF1_HUMAN	K04371	Gallstones:	MK01_HUMAN	K07953	Gallstones:	SAR1A_HUMAN
	Carcinoma:	PROF1_HUMAN		Carcinoma:	MK03_HUMAN		Carcinoma:	SAR1A_HUMAN
K06106	Gallstones:	SRC8_HUMAN	K04391	Gallstones:	CD14_HUMAN	K08517	Gallstones:	SC22B_HUMAN
	Carcinoma:	SRC8_HUMAN		Carcinoma:	CD14_HUMAN		Carcinoma:	SC22B_HUMAN
K06256	Gallstones:	CD44_HUMAN	K04513	Gallstones:	RHOA_HUMAN	K08738	Gallstones:	CYC_HUMAN
	Carcinoma:	CD44_HUMAN		Carcinoma:	RHOA_HUMAN		Carcinoma:	CYC_HUMAN
K07209	Gallstones:	IKKB_HUMAN	K04630	Gallstones:	GNAI2_HUMAN	K10030	Gallstones:	
	Carcinoma:			Carcinoma:	GNAI2_HUMAN		Carcinoma:	**IL8_HUMAN**
K07863	Gallstones:	RHOG_HUMAN	K05765	Gallstones:	COF1_HUMAN	K10159	Gallstones:	TLR2_HUMAN
	Carcinoma:	RHOG_HUMAN		Carcinoma:	COF1_HUMAN		Carcinoma:	
K10030	Gallstones:		K06461	Gallstones:	ITAM_HUMAN	K12799	Gallstones:	
	Carcinoma:	**IL8_HUMAN**		Carcinoma:	ITAM_HUMAN		Carcinoma:	ASC_HUMAN
K12836	Gallstones:	U2AF5_HUMAN	K06464	Gallstones:		K13525	Gallstones:	TERA_HUMAN
	Carcinoma:	U2AF5_HUMAN		Carcinoma:	ITB2_HUMAN		Carcinoma:	TERA_HUMAN
			K10030	Gallstones:		K15464	Gallstones:	BNIP3_HUMAN
				Carcinoma:	**IL8_HUMAN**		Carcinoma:	
			K12799	Gallstones:				
				Carcinoma:	ASC_HUMAN			

*Kyoto Encyclopedia of Genes and Genomes. Bold indicates Interleukin-8*.

**Table 4:** Comparison of human total proteins related to cytokine-cytokine receptor interaction and growth factors and cytokines signaling pathways.

**Cytokine-cytokine receptor interaction** ***N*** **=** **12**			**Growth factors and cytokines** ***N*** **=** **8**		
**Term**	**Condition**	**Protein**	**Term**	**Condition**	**Protein**
K04723	Gallstones:	IL1AP_HUMAN	K05417	Gallstones:	
	Carcinoma:	IL1AP_HUMAN		Carcinoma:	IL11_HUMAN
K05059	Gallstones:	CNTFR_HUMAN	K05424	Gallstones:	LEP_HUMAN
	Carcinoma:			Carcinoma:	LEP_HUMAN
K05060	Gallstones:	IL6RB_HUMAN	K05450	Gallstones:	
	Carcinoma:	IL6RB_HUMAN		Carcinoma:	PDGFD_HUMAN
K05062	Gallstones:	LEPR_HUMAN	K05453	Gallstones:	
	Carcinoma:	LEPR_HUMAN		Carcinoma:	CSF1_HUMAN
K05090	Gallstones:	CSF1R_HUMAN	K05482	Gallstones:	IL18_HUMAN
	Carcinoma:			Carcinoma:	IL18_HUMAN
K05417	Gallstones:		K05511	Gallstones:	
	Carcinoma:	IL11_HUMAN		Carcinoma:	CCL15_HUMAN
K05424	Gallstones:	LEP_HUMAN	K10029	Gallstones:	
	Carcinoma:	LEP_HUMAN		Carcinoma:	CXCL7_HUMAN
K05453	Gallstones:		K10030	Gallstones:	
	Carcinoma:	CSF1_HUMAN		Carcinoma:	IL8_HUMAN
K05482	Gallstones:	IL18_HUMAN			
	Carcinoma:	IL18_HUMAN			
K05511	Gallstones:				
	Carcinoma:	CCL15_HUMAN			
K10029	Gallstones:				
	Carcinoma:	CXCL7_HUMAN			
K10030	Gallstones:				
	Carcinoma:	IL8_HUMAN			

*Kyoto Encyclopedia of Genes and Genomes*.

### Bacterial Protein Analysis

Proportional participation in some taxonomic levels of the imputed protein species is summarized in Table 5. The total protein list was analyzed in KEGG Mapper Reconstruct Pathway focusing on: (1) signaling pathways related to prokaryote interaction, (2) g:Profiler over-represented pathways in human proteins and (3) over-represented pathways in a metagenomic inference analysis (34). The latter analysis was performed from a small microbiota survey within the 20 samples, using bile from the gallbladders of GS patients (*N* = 3), bile from the gallbladders of PDAC patients (*N* = 11) and common biliary brush over the tumor from PDAC patients (*N* = 11) as samples. The results of the analysis show two statistically significant over-represented pathways, pyrimidine deoxyribonucleotide biosynthesis and isoprene biosynthesis (unpublished results).

**Table 5:** Average of the 5 most abundant taxonomic levels per group from identified total bacterial protein.

	**Group**			
	**GS**	**%**	**PDAC**	**%**
Phylum	Proteobacteria	60	Proteobacteria	64
	Firmicutes	15	Firmicutes	13
	Actinobacteria	7	Actinobacteria	6
	Cyanobacteria	4	Cyanobacteria	3
	Bacteroidetes	2,5	Thermotoga	2,8
Class	Gammaproteobacteria	32	Gammaproteobacteria	38
	Alfaproteobacteria	16	Alfaproteobacteria	17
	Bacilli	10	Bacilli	8
	Actinobacteria	7	Betaproteobacteria	6,5
	Deltaproteobacteria	4	Clostridia	6
Order	Enterobacterales	13	Enterobacterales	20
	Bacillales	7	Rhizobiales	6
	Rhizobiales	6,5	Clostridiales	5
	Clostridiales	5	Bacillales	4,5
	Pseudomonadales	4,5	Burkholderiales	4,5
Family	Enterobacteriaceae	8,5	Enterobacteriaceae	13
	Bacillaceae	4,5	Pasteurellaceae	3,5
	Pseudomonadaceae	3,5	Erwiniaceae	3,2
	Geobacteraceae	3	Bacillaceae	3
	Mycobacteriaceae	2,5	Burkholderiaceae	3
Genus	Escherichia	5	Shigella	5
	Bacillus	4	Escherichia	3
	Pseudomonas	3	Bacillus	2,5
	Geobacter	3	Salmonella	2,5
	Mycobacterium	2	Shewanella	2

*GS, gallstones; PDAC, pancreatic ductal adenocarcinoma*.

Upon comparison of the GS and PDAC total protein groups, we found qualitative and quantitative differences regarding quorum sensing, biofilm formation, antibiotic synthesis (biosynthesis of other secondary metabolites) and metabolism of terpenoids and polyketides (Table 6). Regarding metabolism of terpenoids and polyketides, there is a protein involved in zeatin biosynthesis (MIAA_PSECP) that stands out from the other proteins, as it is not present in the PDAC group, is not a protein with an antibiotic function and is specific to that metabolic pathway. Concerning the PDAC group, in the signaling pathway of terpenoid and polyketide metabolism, there is one protein related to surfactin biosynthesis (SRFAB_BACSU), which is also notable, since this protein is also involved in the quorum-sensing signaling pathway. The proteins involved in quorum sensing and biofilm formation show qualitative differences, and the number of proteins present in antibiotic biosynthesis are considerably higher in the PDAC group compared to the GS group. The analysis of bacterial proteins present in the three g:Profiler over-represented human protein signaling pathways related to bacterial infection in the total PDAC group, show no differences in KEGG Mapper Reconstruct Pathway.

**Table 6:** Comparison of bacterial list of total proteins in prokaryote interaction signaling pathways and microbiota metagenomic inference over-represented pathways.

**Metabolism of terpenoids and polyketides**				
**Protein**	**Gen**	**Phylum**	**Gram**	**Pathway**
**GALLSTONES**				
ISPE_RHOP5	ispE	Proteobacteria	Negative	Terpenoid backbone biosynthesis
ISPG_HAEIE	ispG	Proteobacteria	Negative	Terpenoid backbone biosynthesis
ISPG_SHEON	ispG	Proteobacteria	Negative	Terpenoid backbone biosynthesis
**MIAA_PSECP**	**miaA**	**Actinobacteria**	**Positive**	**Zeatin biosynthesis**
FADJ_PECCP	fadJ	Proteobacteria	Negative	Limonene and pinene degradation Geraniol degradation
TKT1_ECOLI	tktA	Proteobacteria	Negative	Biosynthesis of ansamycins
**CARCINOMA**				
ISPE_RHOFT	ispE	Proteobacteria	Negative	Terpenoid backbone biosynthesis
ISPG_GRABC	ispG	Proteobacteria	Negative	Terpenoid backbone biosynthesis
**SRFAB_BACSU**	**srfAB**	**Firmicutes**	**Positive**	**Nonribosomal peptide structures**
**Biosynthesis of others secondary metabolites**				
**Protein**	**Gen**	**Phylum**	**Gram**	**Pathway**
**GALLSTONES**				
DAPB_STRAW	dapB	Actinobacteria	Positive	Monobactam biosynthesis
**CARCINOMA**				
PROA_LEPBL	proA	Spirochaetes	Negative	Carbapenem biosynthesis
PROA_ALKOO	proA	Firmicutes	Positive	Carbapenem biosynthesis
DAPB_GEOKA	dapB	Firmicutes	Positive	Monobactam biosynthesis
DAPB_STRAW	dapB	Actinobacteria	Positive	Monobactam biosynthesis
DAPA_RHOFT	dapA	Proteobacteria	Negative	Monobactam biosynthesis
PGCA_BACSU	pgcA	Firmicutes	Positive	Streptomycin biosynthesis
HIS8_WOLSU	hisC	Proteobacteria	Negative	Novobiocin biosynthesis
TRPE_THET8	trpE	Deinococcus	Negative	Phenazine biosynthesis
**Cellular Community—Prokaryotes**				
**Protein**	**Gen**	**Phylum**	**Gram**	**Pathway**
**GALLSTONES**				
SECA_LACAC	secA	Firmicutes	Positive	Quorum sensing
SECA_CLOTH	secA	Firmicutes	Positive	Quorum sensing
YIDC_METCA	yidC	Proteobacteria	Negative	Quorum sensing
LUXS_DESPS	luxS	Proteobacteria	Negative	Quorum sensing, biofilm formation
**CARCINOMA**				
SP0A_CLOBU	spo0A	Firmicutes	Negative	Quorum sensing
SECA_CLOTH	secA	Firmicutes	Positive	Quorum sensing
**SRFAB_BACSU**	**srfAB**	**Firmicutes**	**Positive**	**Quorum sensing**
PTGA_SHIFL	crr	Proteobacteria	Negative	Biofilm formation
TRPE_THET8	trpE	Deinococcus	Negative	Biofilm formation
CHEB2_BURPS	cheB2	Proteobacteria	Negative	Biofilm formation

*Source: Kyoto Encyclopedia of Genes and Genomes. Bold indicates Bacterial proteins included in the carcinogenesis model*.

## Discussion

To the best of our knowledge, this is the first study to compare human and bacterial proteins, using a metaproteomic approach, bile samples exposed to a tumor vs. non-tumor environment in human PDAC and GS patients, respectively. The characterization of a single species protein profile is known as proteomics, while the characterization of a multi-species protein profile is known as metaproteomics (35). The metaproteomic concept has been studied in humans through the characterization of fecal microbiota and the proteins produced by the different local bacterial species, enabling a better comprehension of the local conditions in the gastrointestinal tract (36, 37). In theory, the microenvironment within the biliary tract and the gallbladder will be more resistant to external variation and more accessible for bile retrieval in animal models. For all that, the biliary tract including its reservoir, will be an ideal biological system to evaluate through metaproteomic and microbiota analyses in conjunction, changes related to specific diets, neoplastic conditions, antibiotic use, chemotherapeutic schemas etc.

Finding meaningful biological information from omics' science data sets has been one of the major challenges of science in recent years (38). The relevance of research findings cannot be measured in every biological instance using statistical significance alone, as not all statistically significant results translate into meaningful biological change. Accordingly, in some areas of science, in which we cannot use statistics, or for which we have not developed appropriate tools, we should look for procedural alternatives that at least enable us to explore the real biological value of data sets. In our research, the three g:Profiler over-represented pathways in human proteins show qualitative and quantitative coincidences and differences regarding the presence of certain proteins in the PDAC and GS groups. The detailed analysis of the proteins in each over-represented signaling pathway is the component of the analysis with the greatest importance. Due to the polyfunctionality of bacteria and human proteins, these proteins must be contextualized and analyzed for relevant biological pathways.

### IL-8: Carcinogenesis and Prokaryote Interactions

IL-8 was identified as a common protein in the three g:Profiler over-represented signaling pathways in PDAC human total proteins, associated with bacterial infections. IL-8 is a human chemotactic interleukin of the C-X-C family also known as CXCL8, originally discovered in macrophages (39), but also produced by epithelial cells. The effect of IL-8 depends on its interaction with specific membrane receptors coupled to G proteins CXCR1 (C-X-C motif chemokine receptor 1, C-X-C) and CXCR2 (C-X-C motif chemokine receptor 2) (40). Under physiological conditions IL-8 levels are undetectable, increasing in the presence of other pro-inflammatory cytokines like tumor necrosis factor α (TNFα) and interleukin 1β (41, 42). None of the latter two cytokines were identified in the PDAC or GS group, suggesting that alternative pathways can stimulate IL-8 synthesis.

High levels of IL-8 are described as poor outcome predictors in many malignant neoplasms, including PDAC. The cellular endpoint effects induced by the IL-8 CXCR1/CXCR2 axis, in normal epithelial cells, tumor cells or other cells in the tumor microenvironment, promote cellular survival, proliferation, angiogenesis, and a stem cell phenotype (43, 44). Concordantly, high levels of IL-8 in patients with breast, prostate and lung carcinoma, and melanoma are related to aggressive tumor behavior, due to high proliferation rate, local invasion, angiogenesis, and an increase of a stem cell phenotype and metastasis (45). In the special case of PDAC, high levels of IL-8 are also related to aggressive tumor behavior and poor prognosis, with evidence that includes PDAC cell line models (46), high blood levels in PDAC and cholangiocarcinoma patients (47, 48), and over-expression of IL-8 and its receptors in tumor tissue (49) and inflammatory cells infiltrating the tumor (50).

The biological relevance of IL-8 is not limited to neoplasms; there is a special prokaryote behavior linked to the synthesis of this interleukin. Biofilm formation by bacteria such as *F. nucleatum* and *A. naeslundii*, and not the planktonic form, stimulates the synthesis of IL-8 by human squamous epithelial cells (51). Supporting the latter concept, several surveys have proved that bacteria biofilm not only stimulates IL-8 synthesis by human squamous epithelial cells, but that stimulation is stronger when the biofilm is formed by multiple bacterial species (52, 53). Furthermore, the similarity of some amino acids in the carboxy-terminal region of IL-8 with cecropins, proteins with antibiotic properties, elicit the analysis of the antibiotic properties of IL-8 through the synthesis of synthetic peptides. These synthetic peptides are thought to be physiologically generated from acidic hydrolysis, and effectively have antibiotic properties which vary according to salt concentration and pH (54).

Some of the PDAC-specific proteins associated with IL-8 are also considered as poor outcome biomarkers in the natural history of malignant neoplasms. High levels of CXCL7 in cholangiocarcinoma tumoral tissue are associated with poor tumor differentiation, local lymph node metastasis, and lymphatic/vascular invasion (55). In renal carcinoma, high levels of CXCL7 are proposed as prognostic factors of chemotherapeutic response (56), and in colon cancer are related to poor survival in patients with liver metastasis (57). Similarly, high levels of CCL17 and IL-11 are associated with poor outcome in malignant neoplasms due to aggressive biological behavior regarding local invasion and metastasis (58–61).

### Differences in Prokaryote Interaction Pathways

The metagenomic inference analysis results from the small microbiota group revealed some over-represented pathways. Of special interest is the metabolism of terpenoids and polyketides signaling pathway. This pathway was analyzed using the total list of bacterial proteins, finding qualitative and quantitative differences, among them the presence of zeatin in the GS group and surfactin in the PDAC group. Terpenoids and polyketides are a huge group of substances synthesized by bacteria, fungi, plants, and animals. Zeatin is an isoprenoid derived from adenine with two isoforms, trans and cis, depending on which of the two hydroxyl groups in the lateral chain of isopentenyl is hydroxylated (62). The identified bacterial protein in the GS group participates in the metabolic pathway for *cis*-zeatin synthesis and is specific to this metabolic pathway. Cis-zeatin is a cytokinin that can be produced by multiple bacterial species (63, 64), with just one published piece of research evaluating its activity in tumor cell lines, proving its anti-tumor potential in leukemia cell lines (65).

The ability to produce surfactin is a property of bacteria from the genus Bacillus, and since the discovery of surfactin in 1968 by Arima, this amphipathic lipopeptide has been found to possess several properties (66). Within these properties are those general to all lipopeptides and antibacterial proteins which act upon Gram-positive and Gram-negative bacteria (67, 68), and other anti-inflammatory (69) and anti-viral effects (70). Of interest in our research, the properties associated with biofilm formation are of utmost importance. Regarding biofilm formation, surfactin has a selective effect, primarily inhibitory, over many bacterial species, though to date there is no clear biological explanation for the selectivity of surfactin for biofilm production/inhibition (71–73).

Besides producing essential compounds for survival, bacteria are able to produce and secrete into the environment low molecular weight compounds called secondary metabolites. Within those secondary metabolites are substances with antibiotic properties that, in a specific microenvironment, confer an advantage upon the bacterium producing the antibiotics, reducing the number of competitors (mainly for nutrient acquisition) (74). Bacterial antibiotic synthesis is a phenomenon influenced by the community and denotes a competitive behavior for survival, and is seemingly species-specific (75). In our analysis, the increased number of identified proteins in the antibiotic synthesis pathways in the PDAC group compared to the GS group is remarkable. Based on that fact, we infer a major competition among species in the PDAC group.

The change in bacteria association, from free-living or planktonic to biofilm formation, relies upon many genetic factors and local conditions (76). Biofilm formation is tightly associated in multiple bacterial species with the increase of c-di-GMP (cyclic diguanylate) intracellular levels, which can also be induced by quorum sensing proteins (77). By means of conventional microbiota analysis, we are unable to determine if the identified bacteria are in a biofilm or not. For this reason, we considered that finding different bacterial proteins among the groups (PDAC and GS) related to quorum sensing and biofilm formation is of biological relevance, as these proteins can be involved in the process of bacteria-induced carcinogenesis. Inflammation is considered a starting point of bacteria associated carcinogenesis. However, this physio-pathological mechanism cannot fully explain the development of carcinomas in the gastrointestinal and biliary tract, as inflammatory states constantly occur throughout the human lifespan, and only a few human beings develop malignant neoplasms in the gastrointestinal system. The bacteria-cancer relationship has been viewed in a reductionist manner as simply a pro-inflammatory milieu initiated by bacteria, in line with the hypothesis of inflammation and cancer proposed by Virchow in 1835 (78). Previous studies have proposed that this pro-inflammatory milieu may be initiated by dysbiosis, which is defined as a change in the normal composition of the microbiota. However, dysbiosis has neither fulfilled the expectations nor provided—to date—a reliable molecular explanation for bacteria-induced carcinogenesis (79).

### The Carcinogenesis Model

**Figure 3:**
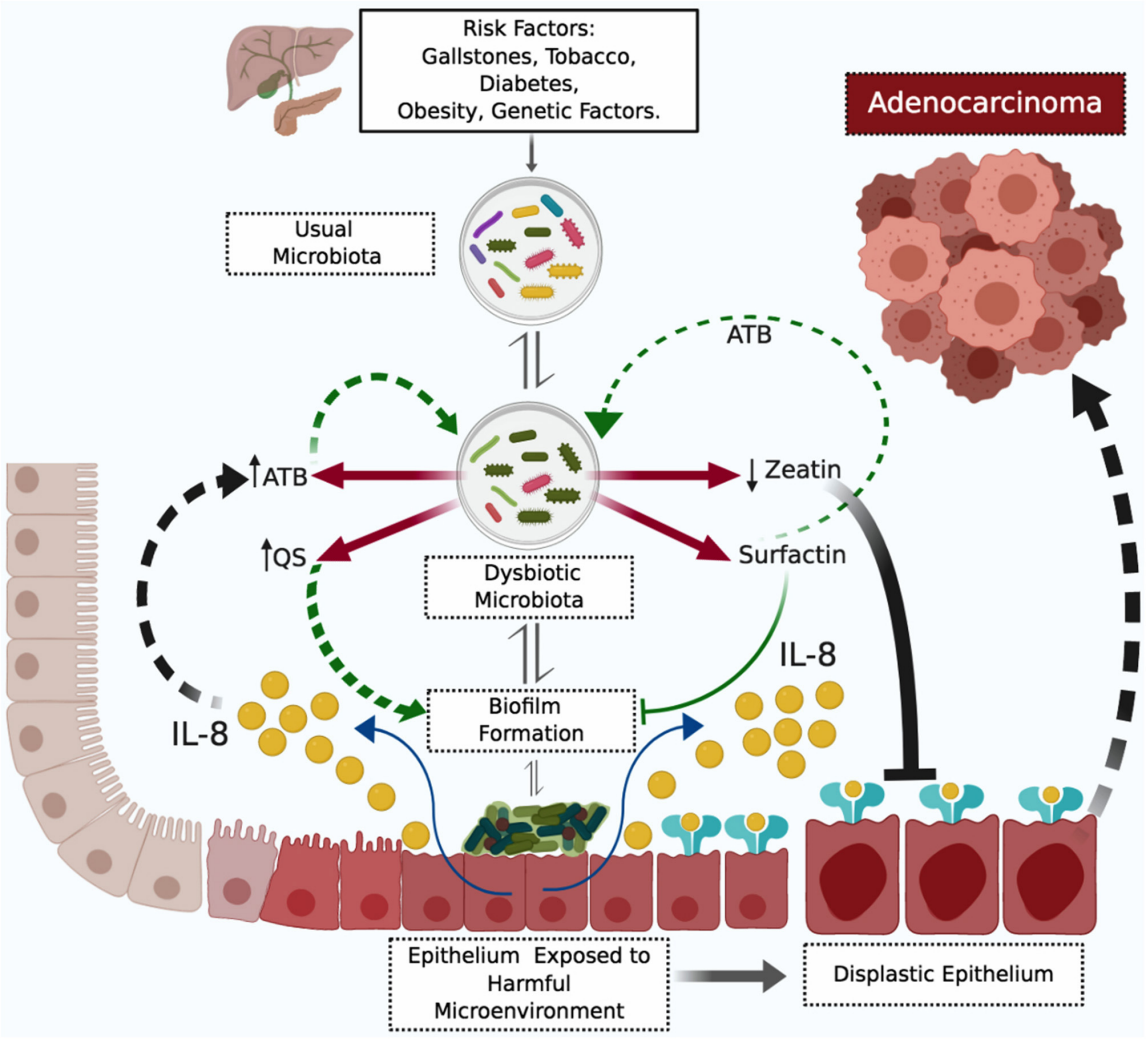
Proposed Model of Biliary Tract Bacterial-Induced Carcinogenesis. The figure depicts a harmful microenvironment originating from prokaryote and eukaryote interaction. The harmful microenvironment initiates with a dysbiotic microbiota product of repetitive inflammatory processes induced by risk factors. The dysbiotic microbiota is specific for low levels of zeatin and high levels of antibiotics (ATB) and surfactin. Surfactin selectively inhibits bacterial biofilm formation—to date—without a molecular explanation for this selectivity. Bacterial biofilm formation stimulates IL-8 (interleukin 8) pro-neoplastic cytokine synthesis by biliary tract epithelial cells. Antibiotics, surfactin, and fragments of IL-8 with antibiotic properties perpetuate the dysbiotic microenvironment. Mutations accumulate in epithelial cells and IL-8 promotes the progression of dysplastic changes to adenocarcinoma, in low zeatin anti-neoplastic protein levels. ATB, antibiotics; IL-8, interleukin 8; QS, quorum sensing proteins.

We hypothesized that there is no such thing as a dysbiotic microbiota, regarding the presence or absence of certain bacterial species. A dysbiotic microbiota is a haphazard composition of bacterial species with products harmful to bacteria and epithelial cells, specific to a particular individual vis-à-vis microbiota-modifying factors. Based on our metaproteomic findings regarding bacterial and human proteins, and its associations, we proposed a biliary tract carcinogenesis model. We are aware of that our proteomic analysis is a snapshot of established PDAC cases, and the propose bacteria-induced carcinogenesis model for the biliary tract is still speculative, not validated, and therefore must be interpreted with caution (Figure 3). The model initiates with unique or multiple dysbiotic factors that promote repeated inflammatory events (80), classically described as stones in the biliary tract, tobacco use, obesity, diabetes mellitus or genetic factors (81). Those promoting factors change the usual biliary tract bacteria composition expected for that individual, shaped according to diet, genetic background, sex, race, age, etc. Promoting factors can create many unusual microbiotas for that individual; though the specific carcinogenic dysbiotic microbiota has a reduced diversity as a sign of competition fostered by highly elevated synthesis of antibiotic products, and qualitative and quantitative differences in bacterial proteins associated with quorum sensing and biofilm formation, such as zeatin and surfactin. High levels of antibiotics maintain the dysbiotic environment, added to the antibiotic effect of surfactin, the latter also selecting, through inhibition, bacteria for biofilm formation. Bacterial species capable of biofilm formation will promote the synthesis of IL-8 by biliary tract epithelial cells. Fragments of IL-8 with antibiotic potential also contribute to maintaining dysbiosis, while the whole protein exerts its pro-neoplastic function of epithelial cellular survival, proliferation, angiogenesis, invasion and stem cell phenotype. The described scenario in conjunction with low levels or absence of zeatin, an anti-neoplastic protein, facilitates the progression of epithelial changes from low-grade dysplasia to adenocarcinoma, through mutation aggregation (82–84). The dysbiotic and harmful epithelial microenvironment needs to continue for a long but unspecified period of time to transform a benign epithelial cell into a malignant one, a period in which the molecular characteristic may be detected.

There are no clear indications for sample size calculations in proteomics research, and the results from a specific protein extraction method, imputation pipeline, and bioinformatic analysis must be validated in further *in vitro* and *in vivo* investigations. Microbiota analysis and dysbiosis alone have not answered the question of the bacteria-induced pathology model. Future research may concentrate on improving the throughput of protein identification from complex biological fluids like bile and consider a combined microbiota and metaproteomic approach to analyze bacterial communities and bacterial and human proteins. It is necessary to start thinking of a change in the dysbiosis paradigm, as we hypothesized dysbiosis is not a specific bacterial composition but rather a harmful protein microenvironment that can be created by several “dysbiotic” microbiotas.

## Data Availability Statement

The datasets presented in this study can be found in online repositories. The names of the repository/repositories and accession number(s) can be found below: ProteomeXchange Consortium via the PRIDE partner repository with the dataset identifier PXD020151.

## Ethics Statement

The studies involving human participants were reviewed and approved by Ethical Committee—Universidad CES. The patients/participants provided their written informed consent to participate in this study.

## Author Contributions

AA and NC-C design the study. DD, OP, and AA participated in sample acquisition. MS-J, NC-C, and AA participated in the laboratory procedures, protein analysis, and biological pathways interpretation. AA and NC-C wrote the manuscript. All authors contributed to the article and approved the submitted version.

## Conflict of Interest

The authors declare that the research was conducted in the absence of any commercial or financial relationships that could be construed as a potential conflict of interest.
